# The emergence of leaders and followers in foraging pairs when the qualities of individuals differ

**DOI:** 10.1186/1471-2148-8-51

**Published:** 2008-02-18

**Authors:** Sean A Rands, Guy Cowlishaw, Richard A Pettifor, J Marcus Rowcliffe, Rufus A Johnstone

**Affiliations:** 1 Department of Zoology, University of Cambridge, Downing Street, Cambridge CB2 3EJ, UK; 2 Institute of Zoology, Zoological Society of London, Regents Park, London NW1 4RY, UK; 3 Centre for Behavioural Biology, School of Clinical Veterinary Science, University of Bristol, Langford, North Somerset BS40 5DU, UK

## Abstract

**Background:**

Foraging in groups offers animals a number of advantages, such as increasing their likelihood of finding food or detecting and avoiding predators. In order for a group to remain together, there has to be some degree of coordination of behaviour and movement between its members (which may in some cases be initiated by a decision-making leader, and in other cases may emerge as an underlying property of the group). For example, behavioural synchronisation is a phenomenon where animals within a group initiate and then continue to conduct identical behaviours, and has been characterised for a wide range of species. We examine how a pair of animals should behave using a state-dependent approach, and ask what conditions are likely to lead to behavioural synchronisation occurring, and whether one of the individuals is more likely to act as a leader.

**Results:**

The model we describe considers how the energetic gain, metabolic requirements and predation risks faced by the individuals affect measures of their energetic state and behaviour (such as the degree of behavioural synchronisation seen within the pair, and the value to an individual of knowing the energetic state of its colleague). We explore how predictable changes in these measures are in response to changes in physiological requirements and predation risk. We also consider how these measures should change when the members of the pair are not identical in their metabolic requirements or their susceptibility to predation. We find that many of the changes seen in these measures are complex, especially when asymmetries exist between the members of the pair.

**Conclusion:**

Analyses are presented that demonstrate that, although these general patterns are robust, care needs to be taken when considering the effects of individual differences, as the relationship between individual differences and the resulting qualitative changes in behaviour may be complex. We discuss how these results are related to experimental observations, and how the model and its predictions could be extended.

## Background

Animals have to make a number of choices when they forage – they need to obtain enough food to ensure that they can survive and reproduce, and do so in a way that ensures they are as safe as possible. A vast body of theoretical and empirical work has been published examining these foraging decisions [1,2], considering both static cases where the animal always makes the same decision about what behaviour it should conduct, and dynamic cases where the behaviour of the animal may change in response to time, environmental cues, and its own energetic reserves [3]. When the animal is foraging, its behavioural decisions will be affected by the amount of food it gains, but other factors may also be important, such as the danger of predation [4-10].

For animals living in social groups, the decisions made by an individual about foraging are further complicated by the actions of the other individuals within the group [11,12]. It may be safer to forage when other members of the group are foraging, since both increased predator detection and dilution of risk [13] mean that individuals may then be able to spend a larger amount of time foraging rather than conducting anti-predator behaviours [14]. However, foraging in groups also brings disadvantages, such as competition for food [15-18] or an increase in predator attacks [19]. The study of social foraging [12,20] asks why animals should forage together, and how this affects group size [21-24] and the rôles played by individuals within these groups [25].

Despite this interest in social foraging, little attention has been given to how foragers decide on their own behaviours within a foraging group, and how these behaviours relate to those of fellow group-members. It is known that many wild and domesticated species of mammals and birds synchronise their activities within foraging groups, such that a majority of the group initiate and conduct the same activity (such as grazing) at the same time. This has been demonstrated for the start and continuance of foraging behaviour in primates [26-29], ungulates [30-45], rodents [46,47] and birds [48-54], and in the foraging-dive behaviour in birds [55-63]. Foraging synchronisation has been suggested to occur in order to increase group cohesion, or to reduce risk of predation [reviewed in [42,43]], but little theoretical work has been done to consider how and when it should occur [but see [64] for a model that considers how synchronisation in leaving time is linked to the level of communication shown between individuals].

Furthermore, if groups are synchronised we are interested in whether all the individuals within the group are behaving identically, or whether individuals are behaving differently to each other, but still producing an overall synchronisation of activities or decisions within the group. We must ask whether group decisions are initiated by certain individuals, and what are the special properties of these individuals that make them more likely to make decisions that are important to the group. At one extreme, a single individual may possess unique qualities that are specific to the decision-making rôle (such as in baboons, where a single dominant male tends to lead the group between sites [65,66]), whilst at the other, essentially identical individuals may consistently make the group's decisions over a period of time (such as in plains zebra, where decision-making females within groups were found to have no specific qualities other than temporarily high energetic requirements due to lactation [67]). A dynamic game by Rands *et al*. [68] exploring activity synchronisation within foraging groups noted that when the actions of a pair of animals that are identical in their energetic requirements and response to environmental parameters are considered, stable differences can emerge between the individuals in their energetic reserves and the rôles they perform within the pair: one individual may consistently make the decisions about how the pair should forage. From this result, Rands *et al*. argued that one individual can consistently act as a leader without possessing any special qualities predetermining its role.

To date, whilst the theory of social foraging has been developed using a variety of analytical and game theoretical techniques, most of the models produced have been static. In this paper, we develop a dynamic game for a pair of individuals, foraging together, each facing a trade-off between starvation and predation risk. State-dependent models of foraging in groups have been developed previously [12,69-74], and dynamic games have shown that diurnal social foraging behaviour [75], social structure [76], and information centre use [77] can be modelled using a producer-scrounger dynamic game framework. However, in the model described by Rands *et al*. [68], each of a pair of individuals was able to base its actions upon its own energetic reserves as well as the actions of its co-player. The model presented by Rands *et al*. [68] was a simple dynamic game which did not consider differences in foraging ability or energetic requirements between pair members, which could potentially affect the ability of the players to synchronise, and the ability of a weaker individual with low energy reserves to make foraging decisions. In the current paper, we expand on these results to consider a more general case. We explore how changing the qualities of identical players (such as differences in foraging ability or susceptibility to predation) can lead to changes in behavioural properties of the players such as their likelihood of foraging, as well as measures of their likelihood of relying upon the other player to make decisions. We then expand on these models to consider players that can differ in these qualities, to consider whether any quality might predispose an individual to the leadership rôle. Using the model, we examine the effects that the environment and predation risk consequently have upon the synchronisation of activity.

## Results

In describing our results, we begin by considering the effects of the various parameters on a pair of players that are identical in their risk of predation, energy reserves, metabolic costs and likelihood of finding food in the environment. We then go on to consider cases where the pair of animals are not identical, and can differ in one or more of these factors.

### General results

**Figure 1:**
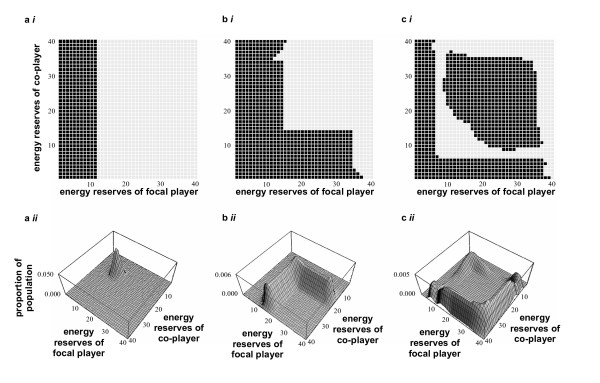
**Examples of policies and population state distributions for identical players. a**) Typical policy (*i*) and stable population distribution (*ii*) for an individual where there is no advantage to foraging with a co-player. The policy shows the optimal behaviour for an individual when its own energetic reserve values and those of its coplayer are known, where a black square means the focal individual should 'forage' during the period, and a white square means that it should 'rest'. (predation risk when foraging alone, *m*_*A*_, is equal to the predation risk when foraging with a co-player, *m*_*T*_: *m*_*A *_= *m*_*T *_= 0.0005; all other parameters set with defaults values as detailed in table 3). **b**) Typical policy (*i*) and stable population (*ii*) where there is an advantage to foraging together: parameters as in table 3; **c**) another form of policy (*i*) and its associated stable population (*ii*) where there is an advantage to foraging together, where symmetry of policy in the central area round the main diagonal ensures that players are synchronised: parameters as in table 3, except *c*_*max *_= 3 state units, *m*_*A *_= 0.0004, *m*_*R *_= 0.00002, *m*_*T *_= 0.00012.

The general results follow the rules described by Rands *et al*. [68], which we redescribe in more detail here as they are crucial to later understanding (and we give much greater consideration to the case where there is a distinct *dis*advantage to foraging together). Examples of the forms of patterns seen are given in Figure 1. If players are identical and there is no advantage to an individual in foraging at the same time as its co-player, then the decision the individual makes is not affected by the energy reserves of the co-player (giving a synchrony coefficient of *D*' = 0). This is seen in the optimal policy of an individual (Figure 1ai), where behaviour is not related to co-player state: if the individual's energy reserves are below a threshold value, it forages in order to avoid starvation; if they are above this threshold, it rests, to avoid exposing itself to a higher predation risk. Therefore, energy reserves should stay near this threshold, as can be seen in the distribution of energy reserves in a stable population of individuals following this policy (Figure 1aii).

If foraging alone is more dangerous to the individual than foraging with a co-player, the optimal behaviour of an individual is dependent upon the reserves of itself and its co-player (examples of policies are shown in Figs. 1bi and 1ci). Figure 1bi shows the simplest (and, from simulations, most likely) policy possible. For the policy shown, behaviour is highly synchronised, giving *D*' = 0.9985. Other policies are more complex, and may specify that the focal individual should forage if its state and that of its co-player fall at certain values within the central region of the policy diagram (such as Fig. 1ci, where *D*' = 1).

The distribution of population energy reserve pairs for policies where there is an advantage to foraging together (Figs. 1bii and 1cii) follows the thresholds at which the players switch behaviours (which can be seen as the edges of the central region of the policy). In the simplest case where players always rest when reserves fall in this central region (Fig. 1bii), the state distribution follows an 'L'-shape. Looking at the likelihood of paired reserve levels in a stable population, the correlation between the reserve pairs is negative (correlation coefficient = -0.589). This means that when one player has low reserves, its co-player should have high reserves. As described by Rands *et al*. [68], the behaviour of the player with low reserves should be dictated solely by its own energy reserve, regardless of the state of its co-player. Because *D*' ≈ 1, the individual with high reserves will therefore copy the behaviour of its co-player. In this situation we therefore expect to see the states of the players distributed so that one player has low reserves, and one with high reserves, and behaviour (determined by the individual with low reserves) should be synchronised.

Where the central region of the policy is more complex, the stable population distribution also becomes more complex (Fig. 1cii), but the energy reserves of the players still follow the edge of the central policy set. In the case shown here, pairs of players are locked into two possible sets of behaviour within the population. A pair could fluctuate around the lower switching thresholds, where one individual is on the lower threshold and its co-player has higher reserves, with swapping of rôles only possible when both individuals reach their lower critical thresholds simultaneously. Similarly, fluctuation could occur around the upper threshold, with rôle-swapping occurring when both players reach their upper threshold simultaneously.

**Figure 2:**
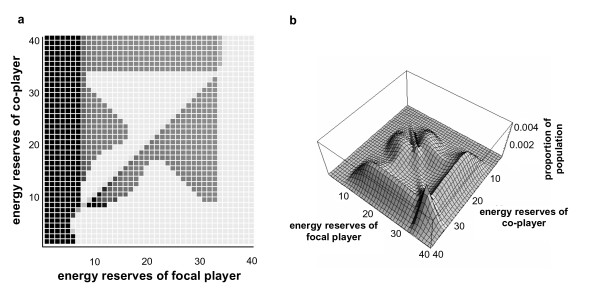
**Policy and population distributions for identical players when there is a disadvantage to foraging together**. This figure shows a case where the risk of being predated when foraging alone is lower than that when foraging together – the optimal policy reflects this by ensuring that players are always engaged in alternative behaviours: **a**) optimal policy, where darkest squares represent 'forage 100% of the time, lightest squares represent 'rest 100% of the time', and intermediate levels of shading represent a continuum between these two. Parameters are as given in table 3, except that *c*_*max *_= 3, *m*_*A *_= 0.00002, *m*_*R *_= 0.00001, *m*_*T *_= 0.00003; **b**) stable population distribution resulting from policy.

It is assumed throughout this paper that foraging together confers an advantage when compared to foraging alone, but we should also consider the case where there are disadvantages to foraging with a co-player, such as through an increased likelihood of detection by predators, or a reduction in mean energetic gain when foraging together [16]. We have investigated these situations, and find that if there is a disadvantage to foraging together, the optimal policy typically resembles Fig. 2a. Calculations show that the behaviours of the pair are highly asynchronous, with a synchrony coefficient value approaching -1 (for the parameter set used in Fig. 2, *D*' = -0.999). The population state-pair distribution is more complex than if foraging together is advantageous: Fig. 2b shows that the distribution of state-pairs tends to follow the behavioural thresholds mentioned above, lending a rectangular shape to the distribution. The paired reserves of the players tend to be randomly distributed (correlation coefficient = -0.05 for the population given in Fig. 2b).

It is possible to synthesise the more general results of the model presented here into a simple behavioural rule of thumb, as described by Rands *et al*. [68]: 'if my reserves are below a set threshold, I should forage; if my reserves are above the threshold, I should rest unless my partner is foraging, when I should also forage' – this describes the 'L'-shaped region that appears in all the policies generated where there is some advantage to foraging together (as is seen in Fig. 1bi). (This is for the case where there is an advantage to foraging together. If it is disadvantageous to forage together, as discussed for Fig. 2, then the rule is more complex and may rely heavily on the calculated policy to determine an individual's optimal behaviour.) It is useful to be able to approximate an individual's behaviour to a simple rule of thumb such as this, rather than a complex policy dependent upon an individual understanding the reserves and various risk and energetic parameters of both itself and a partner: as well as making the optimal behaviour easier to describe, it is easier to understand how a simple rule could be selected for over evolutionary time.

However, as noted above, policies that don't display just an 'L'-shaped region (such as the one shown in Fig. 1ci) can also occur. The rule-of-thumb is followed in the 'L'-shaped region, but there is also a region where the actions of an individual rely upon knowledge of the reserve levels of both players. Arguably, following a policy that includes the ability to judge a co-player's energetic reserves requires more cognitive processing than following the simple rule-of-thumb suggested above. In cases where these extra policy regions should be part of in the optimal strategy, we may still see individuals following a sub-optimal rule-of-thumb that does not include this extra region. A sub-optimal rule-of-thumb nonetheless approximates to the optimal strategy, as pairs of animals following this should never be able to reach a region of state-space where they can behave sub-optimally. Figure 1cii demonstrates this, where a stable population is separated into two groups – those following the 'L'-shaped region of the policy, and those stuck on the edge of the central region. None of the population occur in the intermediate regions of state-space.

### Policy effects of symmetric model

Changes in foraging behaviour in response to changes in parameters follow predictable patterns (Table 1 details all the changes seen in response to systematically altering the parameters: we describe what properties of individuals were measured in detail in the 'statistical presentation' section of the Methods). Increasing the quantity of energy that can be collected in a single period means that foraging is less likely to occur, as it doesn't need to be conducted so often (thereby reducing the player's overall chances of being predated). Increasing either of the metabolic costs means that the player requires more energy, and consequently the likelihood that the player forages increases. Increasing either of the predation risks associated with foraging reduces the likelihood that the player forages, whilst increasing the risk experienced whilst resting means that foraging becomes more likely.

**Table 1:** Changes in behaviours, reserves, and other individual properties when parameters are increased in symmetric models. Trends in properties described come from visual inspection of result sets: '↑↑' denotes a strong tendency for the property to increase in response to a corresponding increase in the parameter investigated; '↑' denotes a noisier but visible increase; '↓↓' denotes a strong tendency for the property to decrease; '↓' denotes a noisier but visible decrease; '-' denotes that the value of the property investigated did not change in response to changes in the value of the parameter; and '×' denotes that changes in the value of the parameter led to unpredictable changes in the property measured.

Property	Changes in property in response to					
	Increase in	Increase in		Increase in predation risk when		
	Energetic gain	Foraging cost	Resting cost	Foraging alone	Foraging together	Resting
Likelihood player forages	↓↓	↑	↑↑	↓↓	↓	↑↑
Likelihood both players forage	↓↓	↑	↑↑	↓↓	↓	↑↑
Likelihood one player forages, the other rests	↓	×	-	-	-	-
Likelihood both players rest	↑↑	×	↓↓	↑↑	-	↓↓
Synchrony coefficient, *D'*	-	-	↑	↑	-	-
Mean energy reserves	↑↑	×	↓↓	↓↓	↓	↑↑
Length of time player repeats behaviour	↑↑	×	↓↓	×	×	↓
Immediate energy reserves difference within pair	↓↓	×	↑	×	×	↑
*C*	↓↓	-	↑↑	↑↑	↓	↓
*S*	↓↓	-	↑↑	↑↑	-	↓

As would be expected, the likelihood of seeing both players resting shows an opposite trend to the likelihood of both foraging, although trends are less pronounced when considering changes in either the cost of foraging (when the effects of changing the parameter are unpredictable) or the predation risk experienced when foraging together (when changing this risk appears to have no effect at all upon resting behaviour).

There is little effect upon the likelihood that both players conduct different behaviours during the period (with a slight decrease in response to an increase in energetic gain), and this is echoed in the relative lack of effect upon the synchronisation coefficient, *D*', due to synchronisation offering such a large benefit to both players.

The changes in individuals' energy reserves mostly follow predictable patterns: increasing energetic gain and its associated increase in resting behaviour leads to there being less need to keep energy reserves high in order to avoid starvation, whilst increasing the cost of resting has the opposite effect. Increasing the cost of foraging has no obvious effect, presumably because of a complex trade-off between the costs and gains directly incurred by the activity. Changes in predation risks do not give obvious trends, apart from an increase in average reserve level in response to an increase in the risk from resting, presumably in tandem with the increase seen in foraging.

There are some effects upon the repetition of behaviours over subsequent periods. Strings of repetitions become longer when energetic gain increases – presumably because players are able to rest for longer periods of time. Similarly, increasing the costs of resting means that players have to swap between resting and foraging more often, although increasing the cost of foraging doesn't have a similar effect. Predation appears to have no obvious effect on the length of time a behaviour is repeated, although making resting more risky reduced the benefits of avoiding foraging for consecutive periods.

The difference in energy reserves between the pair shows the opposite trend to the length of time a player was seen to repeat behaviours. Large differences in reserves mean that the two players are likely to maintain their rôles within the pair for longer, as there is less chance that the player with the larger reserves will lose them through a run of bad foraging periods to the point where rôles are swapped. This suggests that rôles are maintained for longer when players are less likely to repeat the same activity for long periods of time.

The benefits of paying attention to the co-player, as defined by *C *and *S*, falls as energy becomes easier to acquire, but rises when resting becomes expensive (but isn't affected by foraging becoming expensive), presumably because making a mistake and behaving incorrectly becomes more important when metabolic requirements dictate that the player should forage more often. Making a mistake would mean that a player would be more likely to end up foraging on its own, and so increasing the risks associated with that mistake means that *C *and *S *increase, whilst increasing the risks associated with doing the same activity as the co-player leads to a fall in the benefits experienced.

### Policy effects of asymmetric model

Table 2 details all the asymmetric model trends observed. Generalising the results, we found that trends in the properties of player two in response to asymmetries in parameters between the two players tended to (but didn't always) follow those seen in response to similar parameter changes in the symmetric model. However, trends in the properties of player one (whose personal parameters weren't being systematically altered) could not easily be predicted from the symmetric case. We describe these trends in more detail below.

**Table 2:** Changes in behaviours, reserves, and other individual properties when parameters are increased in asymmetric models. The parameters specific to player two were systematically altered as detailed in the methods section. Results given come from visual inspection of result sets: '↑↑' denotes a strong tendency for the property to increase with an increase in the parameter investigated; '↑' denotes a noisier but visible increase; '↓↓' denotes a strong tendency for the property to decrease; '↓' denotes a noisier but visible decrease; '-' denotes that the value of the property investigated did not change in response to changes in the value of the parameter; and '×' denotes that changes in the value of the parameter led to unpredictable changes in the property measured. Note that in the columns referring to changes in player two's metabolic cost of resting and predation risk whilst resting, the parameter value systematically being increased is lower than that of player one: this leads to the difference between the parameter values of the two players becoming smaller as the value of player two's parameter increases. In the other four columns, player two's parameter is greater than that of player one, and so an increase in its value leads to an increase in the difference between the values for the two players.

Property	Changes in property in response to					
	Increase in	Increase in		Increase in predation risk when		
	Energetic gain	Foraging cost	Resting cost	Foraging alone	Foraging together	Resting
Likelihood player one forages	↓↓	↑↑	↑↑	↓	×	-
Player two forages	↓↓	↑↑	↑↑	↓↓	↓	↑↑
Likelihood both players forage	↓↓	↑↑	↑↑	↓	↓↓	↑↑
One forages, two rests	↑	↓	↓↓	-	↑↑	↓↓
One rests, two forages	↓↓	↑	↑	↓	×	↑↑
Both players rest	↑↑	↓↓	↓↓	↑↑	×	↓↓
Synchrony coefficient, *D'*	×	↑	-	↑	↓	×
Mean energy reserves player one	↓↓	↑↑	↑	↓↓	×	×
Player two	↑↑	↓↓	↓↓	↓↓	↓	↑
Immediate energy reserves difference within pair	×	↑↑	↓	↑	-	↑
Length of time player one repeats behaviour	-	-	↓	↑	-	-
Length of time player two repeats behaviour	×	↑	↓↓	↑	-	↓
*C *– Player one	↓↓	↑↑	↑↑	↑	↑	↓↓
Player two	↓↓	↑	↑↑	↑	↑↑	↓↓
*S *– Player one	↓↓	↑↑	↑↑	↑	×	↓↓
Player two	↓	↑	↑	↑	×	↓

Changes in the energetic requirements of player two leads to changes in ndividual foraging behaviour identical to those described for the symmetric model. As well as occurring in player two, these changes also occur in player one, presumably because the advantages of synchronising behaviour with player two. This is reflected in the corresponding increases or decreases in the likelihood of both players resting or foraging at the same time. When the players behave differently during a period, the likelihood that player two is the foraging individual follows the same trend as the likelihood that player two forages regardless of the actions of player one. Changes in the predation risks of player two yield generally similar trends in the behaviours of both players, although these are less discernable in some cases. Similarly, the mean energy reserves of player two reflect those seen in response to the changes made in the symmetric model. Interestingly, although the corresponding changes in player one's energy reserves are mostly straightforward increases or decreases, the trends seen are not immediately predictable from those made by player two.

The synchronisation coefficient *D*' reflects the relationship between proportions of a stable population conducting the four possible pairs of behaviour. The values of *D*' seen don't echo those seen in the symmetric model, and don't reveal much about how parameter changes affect pair behaviour. The only simple trend from a metabolic effect comes where the cost of foraging increases, although it is unclear why this should show a trend whilst the other metabolic effects don't. When the foraging predation risks experienced by player two increase, *D*' either increases (with an increase in predation risk when foraging alone) or decreases (with an increase in the predation risk of foraging together).

The difference in energy reserves between the members of a pair isn't affected by changes in energetic gain, but is affected by metabolic costs: making foraging more costly increases the difference, whilst making resting more costly decreases it. Increasing predation risk whilst foraging alone or resting also leads to an increase in the reserve difference between players. Apart from the latter, none of the trends seen match those seen in the symmetric case, suggesting that there are complex trade-offs involved in determining reserve differences. This is echoed in the lack of similarity seen in the lengths of time that players repeat behaviours for.

The benefits of paying attention to the co-player defined by *C *and *S *follow similar trends to the symmetric model for both players, with the exception of when the cost of foraging increases (where there was an increase the benefits of conducting the correct behaviour when the cost was asymmetric), and when foraging together becomes more dangerous (leading to a similar trend).

## Discussion

These models generate a number of interesting predictions about the behaviour of pairs of animals. Firstly, we confirm the results of Rands *et al*. [68] that behaviour can (and very often should) be highly synchronised, with synchronisation becoming increasingly more apparent when asymmetries between individuals are considered. Furthermore, even if there is no difference between individuals, there is enough complexity in the models to allow a separation of the players into two different rôles (characterised by low or high energy reserves). This affects their subsequent behaviour, their reaction to their co-player, and the ongoing rôle separation between the pair. One important result (or rather, lack of result) from this model is the relative lack of predictable patterns seen in response to asymmetries between the players: given that the modelling assumptions used here about asymmetries were simple and uncomplicated (e.g. one individual uses more energy than its co-player, and nothing else is different), if we were to systematically increase or decrease the asymmetry, we would normally expect a well-behaved model to generate some corresponding increase, decrease, or other recognisable patterns. What we instead saw was, in many cases, something that wasn't easily describable, suggesting that the model assumptions were generating something complex and potentially chaotic (a feasible option given that many simple ecological systems have been shown to lead to chaotic dynamics [78-81]).

### Behavioural synchronisation

The models showed that the emergence of synchronisation is very robust, with synchrony coefficient values near unity in most cases. Therefore, this lends support to the hypothesis [43] that behavioural synchronisation occurs principally because it increases some property of the members of the group (such as a reduction in predation risk, or an increase in energetic gain). But is this realistic? Many experimental studies have shown that synchronisation of foraging behaviour does occur, but it should be noted that in most of these studies, the characterisation of 'synchronisation' is based upon either anecdotal evidence, or simple statistical tests for similarities of behaviour [and there has been debate about the validity of these tests: [82-85]]. Specific techniques for quantifying behavioural synchronisation have been devised by Engel & Lamprecht [86], and used to show synchronisation of behaviour in ungulates [34,41-43]. These studies and a number of other works [87-91] suggest that because the different sexes of large sexually-dimorphic herbivores differ in their digestive and metabolic ability, it is difficult for the two sexes to forage together in synchrony, as their energetic gain would be compromised, and so this may be a contributory factor to why members of these species are usually found in single-sex foraging groups. This could be explored using the rule-of-thumb described in this model, using spatially-explicit simulations of the movements of groups [similar to those described by [92,93]] where individuals differ in their energetic requirements [see also [94,95] for an individual-based model of segregation]. Similar arguments about metabolic requirements may lead to other types of non-heterogeneous assortment of animals, such as those seen in many species of fish, where shoals consist of individuals sharing similar parasite loads, or morphology [96].

In addition to the techniques described above for testing synchronisation, in [68] we introduced the synchronisation coefficient statistic *D*' as a simple means of assessing the proportion of a population that is synchronised at a moment in time. We would caution against its usefulness in experimental studies – particularly because it was near unity in most of the models examined, and because we found few testable trends that would occur in response to environmental manipulation.

There is therefore evidence to suggest that foraging can be synchronised in natural situations, and hence the general predictions of the model may hold. The model also suggests that there should be near-total asynchrony of behaviours if foraging together is less advantageous than foraging alone. However, as discussed by Rands *et al*. [68], it is debatable whether this is a useful result. If social foraging is disadvantageous, then individuals should be selected to forage solitarily. Furthermore, the situation modelled here depends upon members of a pair knowing each other's energetic reserves. If their activities mean that they are separated spatially (as a foraging individual is unlikely to be near a resting individual), then it is unlikely that a strategy dependent upon precise knowledge of a co-player's energetic reserves will evolve as there will be no opportunity to gauge a co-player's energetic reserves during foraging (although it is more feasible if the species is social during some other behaviour, such as sharing a roost or breeding site).

### Rôles of individuals

As well as the synchronisation of feeding behaviour, another interesting property that emerges from the model is both the distinct separation of the energetic reserves of individuals, and the rôles of the two players that emerge with this property. In the simpler of the two policies discussed (Fig. 1bi), we expect to see one individual with low reserves, and one with high reserves, whilst in cases with more complex policies (such as Fig. 1ci), there is still a separation of state into one light individual and one heavy individual, but this is partially dependent upon which set of energy-reserve levels the pair can fluctuate around. The individual whose energy reserves are at a decision boundary (where a fall in reserves will necessitate foraging, whilst a rise will mean resting) will act as the 'pace-maker' for the pair, acting as the individual that decides when the pair should forage. The pace-maker should generally be the lighter of the two individuals if synchronous behaviours occur (for if the individual with the larger reserves is the pace-maker, its co-player is more likely to fall to its lower boundary threshold, where it must forage regardless of its co-player, removing any control over behaviour by the individual with the larger reserves).

The statistics *C *and *S *were proposed as a means of determining the amount of independence a player has with regard to the reserves and behaviour of its co-player [97], where *S *is the more biologically relevant statistic of the two, as it is based upon the benefits to a player of possessing information about its co-player. Because *C *and *S *present similar measures, it is reassuring that the general trends in these that we recorded in our sensitivity analyses are the same. Higher values of either statistic mean that the actions of a player are highly dependent upon those of its co-player. Therefore, we would suggest that leadership behaviour would be more likely to occur when *C *or *S *is high, especially if coupled with a high likelihood of players repeating behaviours over consecutive periods. This would occur in the symmetric case when the resting predation risk was very low.

We predict that we would be more likely to see leadership in non-identical players when there was a large difference in the foraging costs experienced by the two players. Therefore, although leadership can emerge as a property of identical individuals, a noticeable metabolic difference between individuals causes this to be even more likely (although the direction of the changes in *C *and *S *are the same for both players, meaning that we can't easily predict whether the lighter or heavier individual is more likely to become the leader). We also predict that leadership is more likely if the predation risks experienced by either player when foraging alone are similar, although this would be more difficult to experimentally manipulate.

Many vertebrate species have been identified where some form of leadership is identified as occurring (see Leblond & Reebs [98] for a list), and it may well be the case that the leader is a dominant (or experienced) individual rather than a hungry one. Evidence for a 'hungry' individual acting as the pace-maker is seen in swans [48], titmice [99,100], and salmon [101]. Studies of the positioning behaviour of captive and wild roach [102-104] have shown no effect of body size on leadership of the group (where leaders tend to get more food, but suffer an increased predation risk). However, although the initiation of shoal movements was not dependent upon state, once moving, the lead was more likely to be taken by individuals that had been deprived of food. In plains zebra, lactating females (with high energy requirements) were more likely to be the initiators of movement within harems, and no individual consistently acted as the leader [67]. Experiments on captive zebra finches [105,106] showed no relationship seen between either dominance or body mass and leadership status. So, although generally supportive, evidence is ambiguous for whether energetic reserves are important for decision-making in social foragers.

### The effects of pre-existing differences between individuals

The models described here also considered the effects of simple asymmetries between the players. Our principle findings showed that in changing metabolic requirements or predation risk, the effects upon the individual having these parameters changed were broadly similar to those seen in the case with identical players, but the effects upon its co-player were complex, and didn't follow easily quantifiable patterns.

Although the effects of individual asymmetries are not particularly clear, it can be seen that differing abilities of players will have repercussions on their likelihood of conducting certain behaviours, or being in specific states. Understanding how individuals differ within natural populations may allow us to predict their behaviour using these models [92,107]. For example, within dominance hierarchies, individuals may differ in their predation risks, with subordinate individuals having greater risks than dominant ones – perhaps leading to increased vigilance time, or worse access to resources, such as observed by Ekman [108]. Dominant and subordinate individuals can have very different metabolic rates [109] and prey preferences [108,110]. Factors other than dominance also need to be considered: individuals are bound to differ physiologically, and predation risk may differ between the sexes in groups of foraging animals [111]. Also, to maintain a manageable level of simplicity in our model we did not consider the case where there could be an advantage to resting together, which may occur in animals that forage solitarily (such as bats). Similar arguments to those used here to explain why animals forage together have been given for why they should rest together, including predation reduction, and energetic cost reduction through thermoregulation [112,113], and we are confident that future modelling work would show that this also leads to activity synchronisation.

## Conclusion

The model presented here considers a very simple case with just two foragers, but activity synchronisation does seem to appear in the repertoire of socially foraging animals. Through its simplicity, the model allows us to make some basic predictions about state-dependent foraging behaviour, but probably fails to pick up the effects of larger group size [12,75]. Principally, the individuals are constrained to interacting with the other member of their foraging pair, which may not accurately represent what happens in a larger foraging group where individuals can pick and choose who they forage with. In a related paper [92], we extend the rules-of-thumb generated by this model to consider what happens when animals following these rules can interact with more than one individual, and show that although fragmentation of large groups into smaller ones is likely to occur, synchronisation is important within the smaller groups. Empirically, group size may be important in governing behaviour, e.g. sheep in small groups graze for a shorter length of time than those in large groups [114]. Group size may have effects on the number of animals synchronising their behaviour, and bigger groups may see a reduction [62] or increase [55] in their synchrony. Group size will have effects upon the relative predation risk of individuals, and this is known to change patterns of foraging behaviour [115-117].

Further development of state-dependent models may help in answering questions of how groups should behave and how group size should develop [92]. The two-player model we present here is a first step, and the techniques described could be extended to consider larger groups, but at present we are principally limited by the increase in computation time that would be necessary to do this (each new individual considered would cause at the least an exponential increase in the number of state variables to consider, and the rules governing how energetic changes and predation risk change with different numbers of individuals interacting will become much more complex). We would also need to consider how these models related to the cognitive processes of the animal modelled: it is, for example, likely that a foraging animal will be paying attention to more than one neighbour, but the number of individuals it pays attention to will be limited both by its ability to track multiple individuals, and the amount of cognitive processing necessary to both track neighbours and conduct suitable personal behaviour at the same time. It should also be noted that dynamic models need to be tested with care [118], although experimental evidence suggests that this form of modelling can produce ecologically meaningful predictions that are testable [119,120]. Also, in some systems there may be a trade-off between predation risk and starvation, as is modelled extensively in papers by Houston & McNamara [121]. The model presented in this paper could be used to identify the canonical costs of actions in cases such as where predation risk is lower when foraging together, but interference in gain exists.

Although it yielded a number of predictions, the asymmetrical version of the model should be treated with caution when attempting to apply these results to natural populations. As noted, it is necessary to have a good understanding of the exact nature of an asymmetry before it is possible to make predictions, and if animals differ in more than one of the parameters investigated (which is very likely), then opposing behavioural changes are likely to confound what predictions can be made. Furthermore, we must be careful to distinguish which player is the 'heaviest'. Apart from differences in energetic requirements, metabolism, and predation risk, we also need to consider what we mean by 'heavier' individual: in a sexually dimorphic species where females are smaller than males, a well-fed female will nonetheless be lighter than a starving male, and may even have proportionally larger energy reserves per unit of body weight. Therefore, it is likely that if predictions are to be made sensibly for a particular species, then the biology of the species must be inherent to conditions of the model used.

As discussed earlier, we have also suggested a rule-of-thumb that generates social foraging behaviours that approximate to an optimal policy for an individual to follow. Using both this rule of thumb and abstractions of the trends suggested by the asymmetrical results presented here will allow us to construct models of group foraging where individuals are following simple rules [as demonstrated by [92]]. Individual-based models have been highly successful in revealing and exploring much more complex emergent behaviours at the level of the group [122,123], and using theoretically-derived rules (as opposed to rules which produce an approximation of a behaviour, but which have no biological basis or reasoning underlying their use) within these models should greatly add to our understanding of complex natural systems.

## Methods

### Outline

The model considers the actions of a pair of foraging animals. At consecutive discrete moments in time, each of the animals decides either to spend the time until the next decision period foraging, or to rest for the entire period. Both animals decide at the same moment, and their decision is based upon their knowledge of both their own energy reserves and those of their co-player – the ability of an individual to assess the nutritional state of co-players has been demonstrated in fish [124,125] and rats [126]. If an animal forages, it has some probability of finding food in the environment, where the amount of food present follows a known probabilistic distribution. Both foraging and resting are metabolically expensive, and incur differing average energy expenditures (again, the exact loss in a period is probabilistically distributed). So, if the animal rests, it will lose energy, whereas if the animal forages, it will also use energy (where the average metabolic cost of foraging is greater than that of resting) but also has the chance of replenishing its stores, so on average its reserves will increase.

All the actions that an animal conducts will expose it to predators, and it is assumed that foraging is more (or equally) dangerous than resting. It is assumed however that there is a reduction in risk when foraging with the co-player. In most of the models considered, foraging together entails a predation risk that is intermediate between the risks incurred by resting or foraging alone. Other advantages to foraging together were also explored, *e.g*. where gain rate increased with multiple foragers, but these are not described in detail here as they yield similar results to the reduction in predation when foraging together that is considered in this paper.

From these assumptions about the predation risks and energetic changes incurred by both players conducting a specific activity at a moment in time, we can therefore predict how the states of both players change with synchronous or asynchronous behaviours. Using dynamic programming techniques [121,127-129], we are able to calculate how the behaviours of a pair relate to changes in their fitnesses. Houston & McNamara [121] describe in detail the conceptual framework behind the dynamic game methodology used here.

### Model details

The model focuses on the behaviour of a pair of individuals of two different types (where types can differ in their predation risks, foraging success, or energetic expenditure), over a long series of discrete time steps (in other words, we assume an infinite time horizon). At the start of each time step, both individuals make independent decisions whether to rest or forage (until the next time step). The action of individual *i *is denoted *u*_*i *_∈ {*R*, *F*}, where *R *represents the decision to rest and *F *to forage. Each individual experiences some risk of predation during the current time step, depending on its own decision and that of its co-player. Specifically, the probability of death due to predation is *m*_*aR *_for an individual of type *a *when it rests, *m*_*aA *_when it forages alone (while its co-player rests), and *m*_*aT *_when it forages together with the other player. We assume that for individual of type *a*, 0 <*m*_*aR *_<*m*_*aA *_≤ *m*_*aT*_. Because players of two different types (labelled *a *and *b*) can differ in their predation risks when conducting specific actions, we do not assume any relationship between *m*_*au *_and *m*_*bu*_, *u *∈ {*R*, *A*, *T*}. Note that the variables defined (and parameters used in the figures) are summarised in Table 3.

**Table 3:** Parameters used in model. Default values used to generate the figures, and assumptions made in sensitivity analyses.

Variable	Description	Default values for figures 1 and 3	Default values for sensitivity analyses
*c*_*max*_	Largest cost possible	4.0 state units	3.0 state units
*g*_*max*_	Maximum gain during a period	8.0 state units	6.0 state units
*k*	Error in decision making	0.0000001	0.0000001
*λ*	Population adjustment constant	0.1	0.1
*m*_*A*_	Predation risk when foraging alone	0.00050	--
*m*_*R*_	Predation risk when resting	0.00010	--
*m*_*T*_	Predation risk when foraging together	0.00025	--
*μ*_*F*_	Mean cost of foraging	2.5 state units	--
*μ*_*R*_	Mean cost of resting	1.0 state units	--
*ν*	Mean gain from foraging	5.0 state units	--
*ψ*	s.d. of energetic gain when foraging	2.0 state units	--
*S*	Maximum state possible	40 state units	40 state units
*σ*_*F*_	s.d. of energetic cost of foraging	0.5 state units	0.5 state units
*σ*_*R*_	s.d. of energetic cost of resting	0.5 state units	0.5 state units

Each individual *i *may vary in its state, represented by its energetic reserves *x*_*i*_, which takes integer values between 0 and *X*_*i *_(the individual is assumed to be dead if *x*_*i *_= 0, while reserves cannot rise above *X*_*i*_). Energetic reserves are limited within these bounds. During any given time step, each individual incurs uncertain energetic costs dependent on its choice of behaviour. Specifically, the reserves of an individual of type *a *that adopts action *u *in any given time step is reduced by *c *units with probability *κ*_*a*_(*c*; *u*), where *c *ranges from 0 to *c*_max_. When an individual of type *a *forages during any given time step, its reserves are further increased by *g *units with probability *γ*_*a*_(*g*), where *g *ranges from 0 to *g*_max_. Cost and gain probabilities are based upon discretised normal distributions, so that

$$\begin{array}{lll} \kappa_{a}(c;u)=\frac{N\left(c,\mu_{au},{\left(\sigma_{au}\right)}^{2}\right)}{\sum_{c^{\prime }=0}^{c_{\max}}N\left(c^{\prime },\mu_{au},{\left(\sigma_{au}\right)}^{2}\right)}, & \text{and} & \gamma_{a}(g) \end{array}=\frac{N\left(g,\nu_{a},{\left(\psi_{a}\right)}^{2}\right)}{\sum_{g^{\prime }=0}^{g_{\max}}N\left(g^{\prime },\nu_{a},{\left(\psi_{a}\right)}^{2}\right)},$$

where $N\left(x,\mu ,\sigma^{2}\right)=\exp\left(-{\left(x-\mu \right)}^{2}/2\sigma^{2}\right)/\sqrt{2\pi \sigma^{2}}$, *μ*_*au *_and *σ*_*au *_are the pre-discretised mean and standard deviation of the energetic cost for an individual of type *a *of conducting activity *u*, and *ν*_*a *_and *ψ*_*a *_are the pre-discretised mean and standard deviation of the energetic gain experienced by an individual of type *a *when it forages.

A strategy *π *in this game specifies the probability that individual *i *with energy reserves *x*_*i *_forages in any given time step, given that the reserves of its co-player *j *are *x*_*j *_and that the co-player is also following strategy *π*. This probability will be denoted *π*(*i*, *x*_*i*_, *x*_*j*_). Note that *π *encompasses the paired responses of both an individual of type *a *to an individual of type *b *and of an individual of type *b *to an individual of type *a*. Note also that foraging decisions are thus time-independent, and based only on the current state of both players, rather than their history of past interaction. We seek an evolutionarily stable strategy π*, that maximises the long-term survival of individual *i*, assuming that its co-player *j *also adopts the ESS, and taking into account errors in decision-making as detailed below.

### Solution procedure

π* is calculated using an iterated, damped best-response procedure. For the strategy of player *i*, we begin with candidate strategy *π*_0 _(the particular choice of initial candidate strategy does not affect the final ESS obtained; we assume, however, that *π*_0_(*i*, *x*_*i*_, *x*_*j*_) = 0.5 for all *x*_*i*_, *x*_*j*_). Then, an error-prone best response $\hat{b}$(*π*_0_) to this candidate strategy is calculated as described below, and from this a new candidate strategy *π*_1 _is derived as follows:

$$\pi_{n}=\left(1-\lambda \right)\hat{b}\left(\pi_{n-1}\right)+\lambda \pi_{n-1}.$$

Here, *λ *determines the degree of damping [121,130]. Note that as the two players are not necessarily identical, policy calculation must take into consideration both types of player at each iterative step. The iterative procedure is repeated, generating *π*_2_, *π*_3_, *π*_4_, and so on, until $\hat{b}$(*π*_*n*_) ≈ *π*_*n*_, *i.e*. the best response by either player to the current candidate strategy are approximately equal to the candidate strategy itself, which is deemed to occur when $\max_{i,x_{i},x_{j}}\left|\hat{b}\left(\pi_{n}\left(i,x_{i},x_{j}\right)\right)-\pi_{n}\left(i,x_{i},x_{j}\right)\right|<0.0001$. The resulting strategy is taken to be the evolutionarily stable strategy π*.

### Calculation of best-response strategies

The error-prone best response $\hat{b}$(*π*) to a candidate strategy *π *is obtained using dynamic programming. To derive this time-independent best response strategy we proceed as follows. First, we introduce a finite time horizon, *i.e*. we suppose that the game is played over *T *time steps, where *T *is large (the precise number of time steps is irrelevant). Now let *W*_*i*_(*x*_*i*_, *x*_*j*_, *t*; *π*) denote the maximum attainable probability that an individual of type *i *who is alive at the start of time step *t*, with energy reserves *x*_*i*_, paired with a co-player of type *j *with reserves *x*_*j *_who follows the candidate strategy *π*, will survive until the start of the final time step *T*. By default, we assume that *W*_*i*_(*x*_*i*_, *x*_*j*_, *T*; *π*) = 1 for *x*_*i *_> 0, and 0 for *x*_*i*_= 0. Survival probabilities *W*_*i*_(*x*_*i*_, *x*_*j*_, *t*; *π*), and optimal (error-prone) probabilities of foraging *b*_*i*_(*x*_*i*_, *x*_*j*_, *t*; *π*) at earlier time steps *t *<*T *can then be calculated by backwards iteration as described below, by considering at each step the consequences of the different decisions open to the focal individual. In this way, we obtain a time-dependent best response strategy. We continue with the process of backwards iteration until the state-dependent probabilities of foraging specified by the time-dependent strategy converge on fixed, time-independent values, *i.e*. until *b*_*i*_(*x*_*i*_, *x*_*j*_, *t *- 1; *π*) ≈ *b*_*i*_(*x*_*i*_, *x*_*j*_, *t*; *π*) for all *x*_*i*_, *x*_*j*_, which is deemed to occur when $\max_{i,x_{i},x_{j}}\left|b_{i}\left(x_{i},x_{j},t-1;\pi \right)-b_{i}\left(x_{i},x_{j},t;\pi \right)\right|<0.0001$. The resulting set of foraging probabilities are taken to specify the time-independent best response strategy $\hat{b}$(*π*), since they represent optimal (error-prone) behaviour given an infinite time horizon.

The process of backwards iteration, in which we derive *W*_*i*_(*x*_*i*_, *x*_*j*_, *t*; *π*) and *b*_*i*_(*x*_*i*_, *x*_*j*_, *t*; *π*) from *W*_*i*_(*x*_*i*_, *x*_*j*_, *t *+ 1; *π*), starting with *t *= *T *- 1 and working backwards to lower values of *t*, is carried out as follows. Let *H*_*i*_(*x*_*i*_, *x*_*j*_, *t*; *u*_*i*_, *u*_*j*_) denote the probability that an individual of type *i *who is alive at the start of time step *t*, in state *x*_*i *_(> 0), paired with a living co-player of corresponding type *j *in state *x*_*j *_(> 0) who follows the candidate strategy *π*, will survive until the start of the final time step *T*, if it adopts action *u*_*i*_and its co-player adopts action *u*_*j *_in the current time step (assuming that the focal individual *i *thereafter behaves so as to maximise its chances of surviving until time step *T*, taking into account errors in decision making). We then have

$$\begin{array}{lll} H_{i}\left(x_{i},x_{j},t;R,R,\pi \right) & = & \left(1-m_{iR}\right)\sum_{c_{i}}\kappa_{i}\left(c_{i};R\right)[m_{jR}W_{i}\left({x^{\prime }}_{i}\left(c_{i}\right),0,t+1;\pi \right)\\ & + & \left(1-m_{jR}\right)\sum_{c_{j}}\kappa_{j}\left(c_{j};R\right)W_{i}\left({x^{\prime }}_{i}\left(c_{i}\right),{x^{\prime }}_{j}\left(c_{j}\right),t+1;\pi \right)]\\ H_{i}\left(x_{i},x_{j},t;F,R,\pi \right) & = & \left(1-m_{iA}\right)\sum_{c_{i}}\sum_{g_{i}}\kappa_{i}\left(c_{i};F\right)\gamma_{i}\left(g_{i}\right)[m_{jR}W_{i}\left({x^{\prime \prime }}_{i}\left(c_{i},g_{i}\right),0,t+1;\pi \right)\\ & + & \left(1-m_{jR}\right)\sum_{c_{j}}\kappa_{j}\left(c^{\prime };R\right)W_{i}\left({x^{\prime \prime }}_{i}\left(c_{i},g_{i}\right),{x^{\prime }}_{j}\left(c_{j}\right),t+1;\pi \right)]\\ H_{i}\left(x_{i},x_{j},t;R,F,\pi \right) & = & \left(1-m_{iR}\right)\sum_{c_{i}}\kappa_{i}\left(c_{i};R\right)[m_{jA}W_{i}\left({x^{\prime }}_{i}\left(c_{i}\right),0,t+1;\pi \right)\\ & + & \left(1-m_{jA}\right)\sum_{c_{j}}\sum_{g_{j}}\kappa_{j}\left(c_{j};F\right)\gamma_{j}\left(g_{j}\right)W_{i}\left({x^{\prime }}_{i}\left(c_{i}\right),{x^{\prime \prime }}_{j}\left(c_{j},g_{j}\right),t+1;\pi \right)]\\ H_{i}\left(x_{i},x_{j},t;F,F,\pi \right) & = & \left(1-m_{iT}\right)\sum_{c_{i}}\sum_{g_{i}}\kappa_{i}\left(c_{i};F\right)\gamma_{i}\left(g_{i}\right)[m_{jT}W_{i}\left({x^{\prime \prime }}_{i}\left(c_{i},g_{i}\right),0,t+1;\pi \right)\\ & + & \left(1-m_{jT}\right)\sum_{c_{j}}\sum_{g_{j}}\kappa_{j}\left(c_{j};F\right)\gamma_{j}\left(g_{j}\right)W_{i}\left({x^{\prime \prime }}_{i}\left(c_{i},g_{i}\right),{x^{\prime \prime }}_{j}\left(c_{j},g_{j}\right),t+1;\pi \right)] \end{array}$$

where ${x^{\prime }}_{a}(c_{a})=\max(0,x_{a}-c_{a})$ and ${x^{\prime \prime }}_{a}(c_{a},g_{a})=\min(\max(0,x_{a}-c_{a}+g_{a}),X_{a})$.

Averaging over the possible actions of the co-player, the probability ${\bar{H}}_{i}$(*x*_*i*_, *x*_*j*_, *t*;*u*_*i*_, *π*) that an individual of type *i *who is alive at the start of time step *t *and has reserves *x*_*i *_(> 0), paired with a living co-player of type *j *who has reserves *x*_*j *_(> 0), will survive until the start of the final time step *T *if it adopts action *u*_*i *_during *t*, is given by

$${\bar{H}}_{i}\left(x_{i},x_{j},t;u_{i},\pi \right)=\pi \left(j,x_{j},x_{i}\right)H_{i}\left(x_{i},x_{j},t;u_{i},F,\pi \right)+\left(1-\pi \left(j,x_{j},x_{i}\right)\right)H_{i}\left(x_{i},x_{j},t;u_{i},R,\pi \right).$$

A strict best-response strategy would assign a probability of 1 to the action that yields the greatest probability of survival. We follow McNamara *et al*. [131], however, in incorporating errors in decision making, such that there is some probability of adopting the less profitable action. The error-prone, best-response probability of foraging is taken to be

$$b_{i}\left(x_{i},x_{j},t;\pi \right)=\frac{{\bar{H}}_{i}{\left(x_{i},x_{j},t;F,\pi \right)}^{\frac{1}{k}}}{{\bar{H}}_{i}{\left(x_{i},x_{j},t;F,\pi \right)}^{\frac{1}{k}}+{\bar{H}}_{i}{\left(x_{i},x_{j},t;R,\pi \right)}^{\frac{1}{k}}}.$$

Thus, the error-prone best-response strategy assigns equal probabilities to foraging and resting when both yield the same probability of survival, and otherwise assigns a greater probability to the more profitable option (but some non-zero probability to the less profitable course of action). The parameter *k *determines the degree of error: a higher value of *k *implies that choice is less strongly skewed towards the more profitable action, while smaller values of *k *yield strategies that more closely approximate the strict best response.

Finally, having determined *b*_*i*_(*x*_*i*_, *x*_*j*_, *t*; *π*), we can then calculate *W*_*i*_(*x*_*i*_, *x*_*j*_, *t*; *π*) as

$$W_{i}\left(x_{i},x_{j},t;\pi \right)=b_{i}\left(x_{i},x_{j},t;\pi \right){\bar{H}}_{i}\left(x_{i},x_{j},t;F,\pi \right)+\left(1-b_{i}\left(x_{i},x_{j},t;\pi \right)\right){\bar{H}}_{i}\left(x_{i},x_{j},t;R,\pi \right).$$

The calculations required when considering an individual whose co-player is dead are similar in nature to those described above, but simpler, since we do not need to consider alternative possible decisions by the co-player and their consequences. Using the above notation, we have

$$\begin{array}{l} {\bar{H}}_{i}\left(x_{i},0,t;R,\pi \right)=\left(1-m_{iR}\right)\sum_{c}\kappa_{i}\left(c;R\right)W_{i}\left(\max\left(x_{i}-c,0\right),0,t+1;\pi \right),\\ {\bar{H}}_{i}\left(x_{i},0,t;F,\pi \right)=\left(1-m_{iA}\right)\sum_{c}\sum_{g}\kappa_{i}\left(c;F\right)\gamma_{i}\left(g\right)W_{i}\left(\min\left(\max\left(x_{i}+g-c,0\right),X_{i}\right),0,t+1;\pi \right) \end{array}$$

The error-prone, best-response probability of foraging is taken to be

$$b_{i}\left(x_{i},0,t;\pi \right)=\frac{{\bar{H}}_{i}{\left(x_{i},0,t;F,\pi \right)}^{\frac{1}{k}}}{{\bar{H}}_{i}{\left(x_{i},0,t;F,\pi \right)}^{\frac{1}{k}}+{\bar{H}}_{i}{\left(x_{i},0,t;F,\pi \right)}^{\frac{1}{k}}},$$

and *W*_*i*_(*x*_*i*_, 0, *t*; *π*) is given by

$$W_{i}\left(x_{i},0,t;\pi \right)=b_{i}\left(x_{i},0,t;\pi \right){\bar{H}}_{i}\left(x_{i},0,t;F,\pi \right)+\left(1-b_{i}\left(x_{i},0,t;\pi \right)\right){\bar{H}}_{i}\left(x_{i},0,t;R,\pi \right).$$

Lastly, considering a dead individual of type *i*, *W*_*i*_(0, *x*_*j*_, *t*; *π*) = 0 for all *t*, *x*_*j*_.

Using the above series of calculations, we can derive optimal (error-prone) decisions and probabilities of survival at sequentially earlier time steps. As previously stated, we continue to do so until the probabilities of foraging for different state combinations converge on fixed, time-independent values, which are taken to define the (time-independent) best-response strategy.

### Statistical presentation of results

For each set of parameters, a stable behavioural policy was calculated for both individuals, giving the probability for each possible combination of state values that each individual should forage or rest during a period given that it knows both its own state, and that of its co-player. The pair of policies were then used to calculate the distribution of states of player pairs within a stable population. We determined the exact distributions by forward iteration using a Markov chain process [see [127] for details], until the distribution of pairs of states within the population was stable (to allow the population to reach a stability with a non-zero value, we only considered the cases where both individuals survived from one period to the next). We recorded a number of statistical measures in response to parameter changes:

#### 1. Behavioural properties

For foraging behaviour, we consider both the probability of an individual foraging regardless of the behaviour of its co-player (based on the proportions of individuals conducting the behaviour in the stable population), and also consider the more specific cases where either both players are foraging, or where the focal individual is foraging, and its co-player is resting. We also calculated the probability of seeing both individuals resting, and, in the asymmetric case, the probability that the focal player was resting whilst its co-player foraged. We also calculated the average number of times a player repeated a behaviour for. This was done using the Markov chain forward iteration technique, initially noting the expected behaviours of a stable population at a moment in time, and tracking the expected behaviours of the population in subsequent turns: using this, we were able to ascertain how many subsequent periods were necessary before a given proportion of the population had switched at least once in the behaviour they were conducting. We recorded the periods at which at least 50%, 75%, 90% and 95% of the population had switched behaviour at least once away from their initial focal behaviour.

#### 2. Synchronisation

We calculated the likelihood of an individual or pair of players in a stable population conducting a specific behaviour or pair of behaviours in any given time step. The latter was used to calculate a 'synchrony coefficient' *D*' [based on the concept of linkage disequilibrium from population genetics – see [132,133]], calculated as *D*' = (*p*_*RR*_*p*_*FF *_- *p*_*RF*_*p*_*FR*_)/(*p*_*RR*_*p*_*FF *_+ *p*_*RF*_*p*_*FR*_), where *R *= 'rest' and *F *= 'forage', and *p*_*uv *_is the proportion of the population tracked where the focal player conducts behaviour *u *∈ {*R*, *F*} and its co-player conducts behaviour *v *∈ {*R*, *F*}. Using this statistic, *D*' = +1 indicates complete synchronisation of behaviours (where all the members of the population tracked carry out the same behaviour as each other 100% of the time), and *D*' = -1 indicates complete asynchrony (where one player is always foraging and the other is always resting).

#### 3. Measures of sustainability of differences between individuals

We were also interested in how long differences between the players are sustained: we calculated the mean difference between the energy reserves of the two players during any given period of time, and the average number of periods that the player with the higher energy reserves at a given moment in time remained the player with the higher reserves. The general results described by Rands *et al*. [68] demonstrated that individuals within the pair may could display greatly different properties, even when the parameters describing their energetic expenditures and predation risks were identical (described in more detail below): in order to quantify how much the actions of one individual are determined by the behaviour and state of its co-player, we use two statistics [described in detail in [97]]: *C*, which is based upon the fitness cost to an individual of conducting the wrong behaviour when its co-player is in a specific state, and *S*, which is based upon comparing an individual's uncertainty of performing the correct behaviour when it either does or does not possess information about its coplayer's state. Because *C *and *S *should yield broadly similar results, we justify using both here as a means of testing these statistical measures out with a large data set (although in interpreting the statistics, *S *is more deeply grounded in the biological reality of this system, as it is based upon the value of information to an individual, and so we would follow [97] in using this statistic in preference to *C*).

All solutions to the games and their subsequent simulation were calculated using a program coded in C++, using the highest degree of precision possible [134].

### Sensitivity analyses

For both the symmetric and asymmetric cases, sensitivity analyses were conducted to individually explore the effects of the principle parameters used in the model. For the symmetric models where both players were identical, we examined six cases where either *ν*, *μ*_*F*_, *μ*_*R*_, *m*_*R*_, *m*_*A *_or *m*_*T *_was systematically altered. For the asymmetrical models where players 1 and 2 could potentially differ in their properties, the principal parameters were *ν*_1_, *ν*_2_, *μ*_1*F*_, *μ*_2*F*_, *μ*_1*R*_, *μ*_2*R*_, *m*_1*R*_, *m*_2*R*_, *m*_1*A*_, *m*_2*A*_, *m*_1*T *_and *m*_2*T*_; we systematically investigated the six possible single asymmetries where players 1 and 2 differed in the value of one of these parameters (so, for example, *ν*_1 _≠ *ν*_2_, but *μ*_1*F *_= *μ*_2*F*_, *μ*_1*R *_= *μ*_2*R*_, *m*_1*R *_= *m*_2*R*_, *m*_1*A *_= *m*_2*A*_, and *m*_1*T *_= *m*_2*T*_).

For each of these twelve cases, 500 randomised parameter sets were used. These were created by initially generating six pseudorandom numbers *r*_*i *_(where *i *∈ {1, 2, ..., 6} and *r*_*i *_is uniformly distributed within the range [0, 1]). Using these, six temporary values of the initial parameters were calculated:

$$\begin{array}{l} \gamma^{\prime }=4r_{1}+1\\ {m^{\prime }}_{A}=e^{-25r_{2}}\\ {m^{\prime }}_{T}=\left(1-{\left(r_{3}\right)}^{2}\right)\cdot {m^{\prime }}_{A}\\ {m^{\prime }}_{R}=\left(1-{\left(r_{4}\right)}^{2}\right)\cdot {m^{\prime }}_{T}\\ {\mu^{\prime }}_{F}=r_{5}\cdot \gamma^{\prime }\\ {\mu^{\prime }}_{R}=r_{6}\cdot {\mu^{\prime }}_{F} \end{array}$$

(for the asymmetric cases, the equivalent parameters for the two players were initially assumed to be identical: for example, ${\mu^{\prime }}_{1F}={\mu^{\prime }}_{2F}$). Having defined these temporary values, the five parameters that were not being investigated were set equal to these temporary values (for example, in investigating the effects of systematically changing *γ*, we set $m_{A}={m^{\prime }}_{A},m_{T}={m^{\prime }}_{T},m_{R}={m^{\prime }}_{R},\mu_{F}={\mu^{\prime }}_{F}$ and $\mu_{R}={\mu^{\prime }}_{R}$, whilst the parameter being systematically investigated took one of the following values in the symmetric models:

$$\begin{array}{ll} \gamma =\gamma^{\prime }+a_{1}\left(5-\gamma^{\prime }\right)/10, & a_{1}\in \{0,1,...,9\}\\ \mu_{F}={\mu^{\prime }}_{F}+a_{2}\left(\gamma^{\prime }-{\mu^{\prime }}_{R}\right)/2, & a_{2}\in \{0,1,...,9\}\\ \mu_{R}=a_{3}\cdot {\mu^{\prime }}_{R}/10, & a_{3}\in \{0,1,...,9\}\\ m_{A}=\min\left(1,{m^{\prime }}_{A}\left(a_{4}+1\right)\right), & a_{4}\in \{0,1,...,9\}\\ m_{T}={m^{\prime }}_{T}+a_{5}\left({m^{\prime }}_{A}-{m^{\prime }}_{R}\right)/10, & a_{5}\in \{0,1,...,10\}\\ m_{R}=a_{6}\cdot {m^{\prime }}_{T}/10, & a_{6}\in \{0,1,...,10\} \end{array}$$

In the asymmetric models, all the parameters for player one were set equal to the temporary values, and all the parameters for player two apart from the parameter that was systematically altered were set equal to those of player one. The parameters being systematically investigated took one of the following values:

$$\begin{array}{ll} \gamma_{2}=\gamma_{1}+a_{7}\cdot \left(\gamma_{1}-\mu_{1F}\right)/5, & a_{7}\in \{1,2,...,10\}\\ \mu_{2F}=\mu_{1,F}+a_{8}\cdot \left(\gamma_{1}-\mu_{1F}\right)/8, & a_{8}\in \{1,2,...,7\}\\ \mu_{2R}=\mu_{1R}\cdot \left(a_{9}+8\right)/16, & a_{9}\in \{1,2,...,8\}\\ m_{2A}=a_{10}\cdot m_{1A}, & a_{10}\in \{1,2,...,9\}\\ m_{2T}=m_{1T}+a_{11}\cdot \left(m_{1A}-m_{1T}\right)/8, & a_{11}\in \{0,1,...,8\}\\ m_{2R}=a_{12}\cdot m_{1R}/8, & a_{12}\in \{0,1,...,8\} \end{array}$$

## Authors' contributions

The grant was written by and awarded to GC, RAJ, RAP and JMR. The model examining identical individuals was initially created by RAJ, and expanded upon by SAR, who coded the asymmetrical version, conducted the analyses, and wrote the initial draft of the manuscript. The final draft and revisions were read and approved by all the authors.
